# Uncovering Nursing Communication Strategies and Relational Styles to Foster Patient Engagement in Oncology: A Scoping Review

**DOI:** 10.3390/healthcare12131261

**Published:** 2024-06-25

**Authors:** Andrea Francesco Crivelli, Serena Barello, Marta Acampora, Loris Bonetti

**Affiliations:** 1 Department of Business Economics, Health and Social Care, University of Applied Sciences and Arts of Southern Switzerland (SUPSI), 6928 Manno, Switzerland; andrea.crivelli@usz.ch (A.F.C.); loris.bonetti@supsi.ch (L.B.); 2 Institute for Intensive Care Medicine, University Hospital Zurich, 8091 Zurich, Switzerland; 3 Department of Brain and Behavioural Sciences, University of Pavia, Piazza Botta 11, 27100 Pavia, Italy; 4 IRCCS Mondino Foundation, Via Mondino 2, 27100 Pavia, Italy; 5 Department of Psychology, Università Cattolica del Sacro Cuore, 27100 Milan, Italy; marta.acampora@unicatt.it; 6 Nursing Research Competence Centre, Department of Nursing, Ente Ospedaliero Cantonale, 6500 Bellinzona, Switzerland

**Keywords:** communication, neoplasm, nurse, nurse–patient relation, patient engagement, scoping review

## Abstract

Nurses play an active role in fostering engagement of oncological patients, and, therefore, adopting effective communication and interpersonal skills is crucial. However, the nurse–patient relationship and communication strategies are frequently undervalued. This scoping review aims to address this gap with a twofold objective: (1) to explore the existing literature to identify communication strategies and relational styles employed by nurses to promote patient engagement in non-pediatric oncology patients; (2) to assess current knowledge on this topic to determine the need for future research. The search was conducted on different scientific databases and grey literature. The review was conducted following the methodology outlined in the Joanna Briggs Institute guidance for scoping reviews and the updated version of the PRISMA-ScR Checklist. Thirteen articles were included in the study. The studies in total enrolled 863 participants. Four clusters of nursing interventions were identified, encompassing communication strategies and relational styles of varying complexity, along with ten categories of general outcomes emerging from their implementation. This study summarizes the current knowledge regarding nursing communication strategies and relational styles used to promote patient engagement in oncological patients. Further research is needed, to evaluate and integrate the researched techniques, tools, and interventions for future clinical nursing practice.

## 1. Introduction

In the field of oncology, nurses play a vital and active role throughout the entire continuum of cancer care. They are instrumental in facilitating patients’ active involvement in their own healthcare journeys through the application of relational-based care models and communicative interventions. By utilizing these approaches, nurses empower patients to take an engaged role in their health management and decision-making processes, thereby enhancing the overall quality of care in oncology settings [1,2,3,4,5].

Cancer has been a significant global cause of mortality since the early decades of the 20th century. According to Sung et al. [6] in 2020, worldwide, the incidence of new patients with cancer was 19.3 million and mortality was nearly 10 million. The World Health Organization (WHO) [7] identifies cancer as the first or second leading cause of death in people younger than 70 years in 112 out of 183 countries and the third or fourth in the remaining 23. Incidence and mortality are increasing worldwide, reflecting the ageing population, population growth, the distribution of major risk factors often associated with a nation’s socioeconomic development [6], the socio-demographic context characterized by multiculturalism, and the increase in chronic diseases [8,9].

The field of cancer care operates within a complex framework that is continuously evolving. It necessitates an integrated, coordinated, and multidimensional approach involving various disciplines. Healthcare policies and systems worldwide have undergone a significant paradigm shift since the 1960s. This shift marked a major reinterpretation and transformation of the traditional medical and healthcare models that had guided these disciplines for centuries. This evolution has led to the emergence of the era of “participatory medicine”, emphasizing the active involvement of patients in their own healthcare decisions and promoting a collaborative approach among healthcare professionals, patients, and their families. This multidisciplinary and participatory approach is essential in addressing the multifaceted challenges posed by cancer and ensuring comprehensive and patient-centered care [9].

In this era of evolving healthcare models, the concept of patient engagement has emerged as a crucial component. Weil [10] describes patient engagement as an “imperative” that is necessary for this ongoing transformative process. Similarly, Dentzer [11] characterizes patient engagement as the “blockbuster drug of the 21st century,” highlighting its tremendous potential and impact on healthcare outcomes. Patient engagement entails actively involving patients in their own care, empowering them to make informed decisions, and fostering a collaborative partnership between patients and healthcare providers. Recognizing the significance of patient engagement is essential in promoting patient-centered care and improving health outcomes in the 21st century. 

Patient engagement is a comprehensive concept that encompasses various socio-cultural factors and individual characteristics, as well as interpersonal, emotional, social, and organizational elements. It refers to the collective influences that impact an individual’s capacity to become more proactive, informed, and involved in their healthcare journey. Patient engagement goes beyond mere participation and includes factors such as empowering patients to take an active role in their healthcare decisions, promoting health awareness, facilitating effective communication and collaboration between patients and healthcare providers, and fostering a supportive organizational environment. This umbrella term acknowledges the multifaceted nature of patient engagement and recognizes the diverse factors that shape individuals’ ability to actively engage in their healthcare experiences [8,12,13]. In this new paradigm, individuals with a disease are not merely passive recipients of care but active participants who possess their own desires and play a co-authoring and leading role in their healthcare journey. They have the ability to self-determine and navigate their path of care. This concept of patient engagement is increasingly recognized as a vital component of high-quality healthcare services [14] and has become a significant area of research interest [15]. The goals of patient engagement are multifaceted, dynamic, and systemic, as they align with the multidimensional nature of patient engagement as a cognitive, emotional, and behavioral activation process for individuals [8]. These goals include fostering a greater awareness of one’s health condition, empowering individuals to activate the healthcare system when necessary, enabling appropriate responses to disease-related symptoms, and becoming “ambassadors of good practices” [8].

The literature describes various benefits associated with the implementation of patient engagement. These benefits encompass increased personal satisfaction, reduced risks through enhanced prevention of errors and adverse events, improved disease management, enhanced trust in healthcare practitioners and caregivers, improved therapeutic adherence, decreased costs, and improved communication and relationships between patients and healthcare professionals. The implementation of patient engagement holds immense potential for improving healthcare outcomes and experiences for all stakeholders involved [14,15,16,17,18,19,20,21].

Furthermore, studies have found that building a good relationship and communication between patient and nurse is a prerequisite and a pivotal point in the promotion, achievement, and maintenance of patient engagement. As a matter of fact, in the literature nurses play a strategic role in promoting patient engagement and are increasingly recognized as a frontline professional figure who implements relational and communication skills on a daily basis in order to actively involve the patient in care [15,17,18,21,22,23,24,25,26]. Moreover, according to Cortes et al. [27], in order to promote engagement, it is necessary that the patients and their relatives are involved in the therapeutic pathways and are supported with good communication skills. From a nursing perspective, patient engagement is a process that requires giving better and more reliable information to the patient and hands over more control and influence over their unique clinical care pathway. Patients and their families build a mutual partnership with the nurse and their engagement in the care process is fostered [28]. The nurses can thus develop an appropriate care and treatment plan “tailored to the patient”, addressing issues such as symptom management, medication management, or the shared formulation of meaningful discharge goals [14]. In addition, according to Mary Jean Schumann, the nurse is in a privileged and exclusive position to help the patient become more involved in their care pathway [19] and influence the level of patient engagement [29]. To build this mutual partnership and durative relationship, the nurse needs to be competent in their communication and interpersonal knowledge and skills, which is an aspect that justifies and clarifies the importance of investigating the chosen topic.

Indeed, communication holds immense importance in oncology nursing, being recognized as the “golden key” and a necessity for practitioners, patients, and caregivers. However, it is unfortunate that communication and the nurse–patient relationship are often undervalued [30]. Adopting effective communication and interpersonal skills is crucial throughout the entire cancer care continuum, underscoring the need for this literature review. The aim of this scoping review (ScR) is to elucidate the available evidence on communication–relational tools, interventions, and techniques that empower nurses to implement patient engagement in adult patients with cancer. The primary objective is to identify gaps in the current literature’s knowledge and provide guidance for future research endeavors. The review encompasses diverse research designs and explores a wide range of literature to offer a comprehensive mapping of the current understanding of this topic. By doing so, it enables the formulation of hypotheses and the establishment of future research directions on this complex and significant subject matter, which holds great relevance for healthcare systems as a whole. 

Currently, to the authors’ knowledges, no ScRs have been conducted to highlight the knowledge regarding communication strategies and relational styles that the nurse adopts to promote patient engagement in oncology.

## 2. Methods

### 2.1. Guidelines and Research Protocol 

This scoping review was designed according to the updated version of the Preferred Reporting Items for Systematic reviews and Meta-Analyses extension for Scoping Reviews (PRISMA-ScR) Checklist [31] and the Joanna Briggs Institute guidance for scoping reviews [32].

No protocol has been published for this research.

The research question has been developed using the PCC mnemonic (as follows), as well as the exclusion and inclusion criteria.

### 2.2. Research Question

PCC

- Population/Participants: patient with cancer, not pediatric.
- Concept: what communication strategies and/or relational styles does the nurse implement in her clinical practice to promote patient engagement?
- Context: open (any limits).

What types of communication strategies and/or relational styles does the nurse implement to foster patient engagement in non-pediatric patients with cancer?

### 2.3. Inclusion and Exclusion Criteria

#### 2.3.1. Population/Participants

The ScR’s population is represented by all patients with cancer, excluding pediatrics patients. The latter, indeed, require communication strategies and/or relationship styles that greatly differ from those of adult patients. Any other exclusion criteria were applied in terms of clinical, economic, and social conditions, sex, age, and/or ethnicity.

#### 2.3.2. Concept

All studies including nurses (RNs), advanced practice nurses (APNs), nurse practitioners (NPs), oncology nurse specialists, and nursing students were considered for any relational and/or communication intervention aimed at implementing patient engagement in patients with cancer.

The phenomena of interest were the communication strategies and/or relational skills used by nurses in the oncology field to encourage trust, enhance self-management and develop specific clinical skills, and achieve other observable patient outcomes associated with patient engagement.

In particular, the focus is on how patient engagement can favor the improvement of clinical conditions, knowledge of exacerbations or disorders, greater involvement in the active participation in decision making with respect to one’s disease path, disclosure of health costs, the satisfaction experienced by both patients and caregivers following the establishment of the relationship, and the introduction of new types of communication, education, and relationships in certain contexts of care [14,15,16,17,18,19,20,21].

#### 2.3.3. Context

The ScR encompassed all types of communication strategies and relational styles suitable for promoting patient engagement in oncological patients in all contexts of care, including, for example, hospital, ambulatory, home, or rehabilitation settings. No limits were placed on the included studies regarding geographical area, care settings, or cultural context. 

#### 2.3.4. Types of Sources of Evidence

To map the evidence and explore current knowledge on the subject, the following types of sources in the literature are considered in the review:

- Experimental/epidemiological and quasi-experimental studies, such as randomized controlled trials, non-randomized controlled trials, before and after studies, and interrupted time series studies;
- Observational studies, such as cohort studies, case–control studies, analytical cross-sectional studies, case studies, individual case reports, and descriptive cross-sectional studies;
- Qualitative studies, such as phenomenological, ethnographic, descriptive, grounded theory, and action research.

Research protocols, pilot studies, letters to the editor, and secondary studies such as systematic reviews, guidelines, and meta-analyses have been excluded.

### 2.4. Electronic Databases

The following electronic databases were searched: PUBMED, CINAHL, Cochrane library, Ovid Nursing Database, and APA PsycInfo. The last search was completed in November 2023. Moreover, a grey literature search was conducted, especially through Google Scholar, but also in public health websites and specific scientific magazines, using so-called hand searching.

### 2.5. Search Strategy

The search strategy was conducted using the terms “neoplasm” (and related terms), “nurse” (and related terms), “health communication” (and related terms), “nurse-patient relation” (and related terms), and “patient engagement” (and related terms), all previously grouped in a logic grid. Supplementary File S1 reports the completed search strategy for one database. The search strings for the different databases were written and adapted using the specific terms found in the thesaurus and MeSH of each electronic database. In Supplementary File S2 the full electronic search strategy for PUBMED is presented. Duplicates were removed using Zotero [33]. After an initial screening of titles and abstracts, the full texts of those articles that were considered relevant to the research question were read. Finally, the bibliographies of the included articles were evaluated for important missing information. 

### 2.6. Study Selection

The selection of studies was carried out independently by two researchers in two steps. The first step involved reading the titles of the articles, while the second step entailed reading the complete abstracts. Duplicates, which originated from other databases, were identified using the author’s name, title, and the period in which the study was conducted. The papers that met the inclusion criteria were evaluated by two researchers. The decision to include or exclude each study was made collectively after a discussion. Should there have been any discrepancy in the decision to include or exclude a paper, a third researcher would have been consulted. 

**Figure 1:**
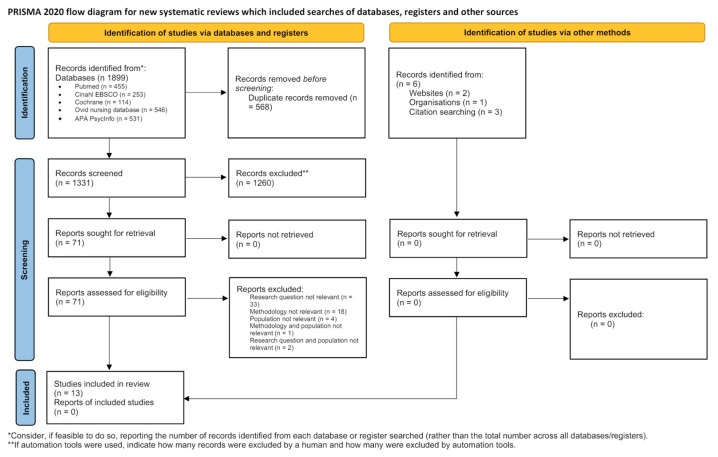
PRISMA Flow diagram [34].

The PRISMA statement flow chart reports the study selection process (Figure 1).

Supplementary File S3 reports the reason for exclusion of the studies not included in this research.

The quality assessment tool by Hawker et al. [35] was used to assess the included papers (Supplementary File S4).

### 2.7. Data Charting Process

The key information extracted from the sources found are those suggested by the JBI manual [32] and are: authors, year of publication, title, country of origin, purposes, population, methodology, type of intervention, outcomes, and key factors related to the research question.

### 2.8. Synthesis of the Included Studies 

The ScR intends to map and summarize the available evidence on the topic under analysis without aiming to modify the current protocols and guidelines for clinical decision making. Therefore, the analysis is a descriptive qualitative content analysis, rather than a thematic analysis/synthesis.

For this analysis, the method of categorizing macro-topics (clusters) found in the literature is used [32]. 

## 3. Results

### 3.1. Selection of Sources of Evidence

First, 1905 articles were identified: 1899 through the databases search and 6 through the manual search. After removing duplicates, 1332 titles and abstracts were screened according to the inclusion and exclusion criteria. A total of 71 articles were assessed eligible for full-text screening and evaluated in their entirety, and, of these, 13 were deemed relevant for analysis. The reasons for exclusion were as follows: did not meet the research question, did not meet the inclusion criteria regarding the methodology, did not meet the inclusion criteria regarding the population, did not meet the inclusion criteria regarding either methodology and population, and/or did not meet the inclusion criteria either research question and population.

The quality of the studies was generally rated as good. See Supplementary File S4 for more details.

The selection process is summarized in Figure 1.

### 3.2. Study Characteristics

Of the thirteen articles included, three are quantitative, six qualitative, two are quality improvement projects, and two are mixed-method. Table 1 shows the characteristics of the included studies. 

#### 3.2.1. Population 

The studies overall enrolled 863 participants. Sample sizes ranged from 8 [36] to 207 [37] participants. All study participants were patients with cancer, 18 years of age or older, were able to make decisions, and were cognitively competent, conscious, and oriented (as specified in most studies). In three studies, the population was specific to a type of cancer: Berger-Högler et al. [38] considers people with ductal carcinoma in situ; Sundberg et al. [39] focuses on prostate cancer; and Sharp et al. [40] focuses the research on patients with head and neck cancer. Other specific populations are related to the type of treatment, prognosis of the disease, or length of hospitalization [41,42,43]. The first study [41] considers only patients admitted for colorectal cancer surgery at a surgical service; the second study [42] includes patients who were not dismissed until 48 h after hospital admission; the third [43] patients with noncurable advanced disease. Finally, the language spoken by study participants also appears to be important as an inclusion or exclusion criteria. Two examples are the articles by Chang et al. [44], in which participants had to be able to speak Taiwanese and/or Mandarin, or that of Sundberg et al. [39], where knowledge of Swedish was required.

#### 3.2.2. Concept

The phenomenon of interest in this ScR is the use of communication strategies and/or relational styles that promote patient engagement. Different types of interventions and results were identified, which are explored in Table 2, Table 3, Table 4 and Table 5. The communicative-relational nursing techniques, tools, and interventions accepted in the literature review are effective in activating and promoting patient engagement (understood as an umbrella concept), as they report expected results and outcomes such as reduced re-hospitalization, perceived satisfaction, or decision-making ability (Table 6). The different types of interventions have been classified into four clusters.

The first cluster classifies less complex interventions that do not require a strong trusting relationship with the patient. Burrows Walters and Duthie [41] highlight the importance of ‘basic communication strategies’, such as the use of understandable terminology and concordance between body language and verbal messages. Moreover, Bottorff et al. [36] identify comforting strategies, which are intrinsic to the nurse–patient relationship, such as providing emotional support or information. Other strategies include the use of written or audio–visual material and written signals to reinforce communication. Effective communication, according to Jenerette and Mayer [24], is crucial for self-management and self-efficacy. An example is the Teach-back method, which involves asking the patient to explain the concept discussed in their own words [47]. This technique, which is easy to implement, according to the Agency for Healthcare Research and Quality, has positive effects on patient engagement [42]. A review by LeBlanc [48] shows that the Teach-back method strengthens information transmission, health literacy, and SDM. Callaway et al. [42] combine Teach-back with bedside handoffs and discharge bundles to enhance communication and patient activation.

The second cluster concerns communication strategies and relational styles—specific information and education. Among the interventions in this cluster are those using ICTs, which are crucial for patient engagement and transparency of health information [8]. ICTs include patient portals, telephony, e-mail, instant messaging, and symptom self-management applications [39,49]. Follow-up phone calls are crucial for building a trusting relationship [45]. ICTs improve transparency, information management, and trust between caregiver and patient [39]. By communicating frequently with patients, nurses can establish supportive relationships and recognize problems that are not apparent with other professionals [45]. ICTs are not only tools, but new communication strategies that enlarge the possibilities of relationship and management. The latter are aspects related to patient engagement that can also be observed in non-ICT educational tools such as care diaries [40], which emphasise effective communication and a feeling of being considered.

The third cluster comprises communication strategies tailored to decision making. The interventions are based on several communication strategies within the same intervention, and the use of durable and structured tools that require time to be learnt. For example, Berger-Höger et al. [38] present an intervention using evidence-based document and nurse-led decision coaching to help patients in SDM. Kawasaki [37] explores consultation techniques for SDM, highlighting the need to share emotions, thoughts, and preferences.

The last cluster includes studies that support and motivate patients in their empowerment and engagement. Walkzak et al. [43] use live and telephone interviews to activate patients in the discussion of prognosis, developing health literacy-related self-efficacy. Chang et al. [44] observe the patient empowerment process through Freire’s dialogical interviewing, emphasizing the building of an empathic and trusting relationship. Mirabella et al. [46] present educational interventions based on SMART goals developed by patients according to their learning needs, and related to their specific care path, their experiences, or educational modules offered by nurses. During the intervention, nurses establish relationships and provide education, using open communication, Teach-back communication methods, and other communication tools. Twibell et al. [15] identify the relationship with the nurse as crucial for patient engagement in fall prevention program, emphasizing the importance of being compassionate, empathic, and interested in the patient’s life story.

#### 3.2.3. Context

The studies cover four continents (America, Europe, Asia, and Oceania) and seven nations (USA, Germany, Taiwan, Japan, France, Sweden, and Australia). They were conducted in a variety of care settings including hospitals, specialty clinics, national cancer research centers, a military hospital, and home follow-ups.

### 3.3. Study Results

#### 3.3.1. Main Communication Strategies and Relational Styles

**Figure 2:**
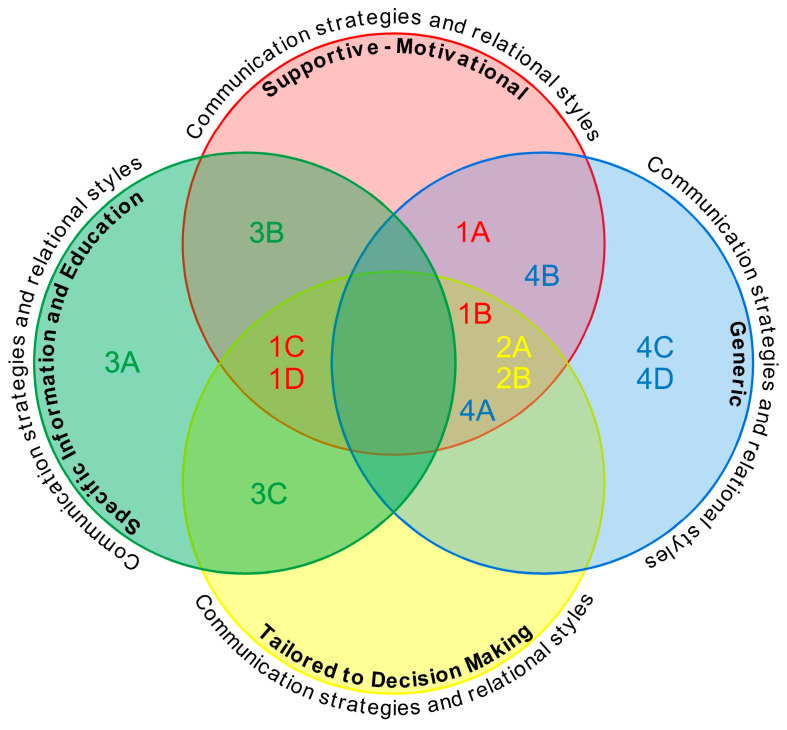
Interaction between the four major dynamic clusters concerning the nursing communication and relational interventions used to implement patient engagement. Communication strategies and relational styles: (1) supportive–motivational; (1A) establishment of a relationship of trust; (1B) Freire’s dialogical method—dialogical interviewing; (1C) SMART educational intervention; (1D) communication support program; (2) tailored to decision making; (2A) consultation techniques in SDM; (2B) decision coaching; (3) specific information and education; (3A) care diaries; (3B) telephone follow-up (ICT); (3C) using an application on smartphones and tablets (ICT); (4) generic; (4A) multifaceted approach; (4B) comforting nursing strategies; (4C) basic communication strategies; (4D) Teach-back method.

The research identified several nursing communication and relational interventions that have been used in practice to implement patient engagement or related aspects of this umbrella concept. Four clusters were created. These clusters, however, are not to be understood as watertight compartments that do not communicate with each other. They group together those interventions with the greatest predisposition to that category, which may also have similarities or related characteristics to the others. This aspect can be observed in Figure 2, which—through a graphical representation—shows the position of the different interventions identified in the studies in relation to the four categories created.

1. Communication strategies and relational styles—supportive–motivational.
  A. Establishment of a relationship of trust.
  B. Freire’s dialogical method—dialogical interviewing.
  C. SMART educational intervention.
  D. Communication support program.
2. Communication strategies and relational styles—tailored to decision making.
  A. Consultation techniques in SDM.
  B. Decision coaching.
3. Communication strategies and relational styles—specific information and education.
  A. Care diaries.
  B. Telephone follow-up (ICT).
  C. Using an application on smartphones and tablets (ICT).
4. Communication strategies and relational styles—generic.
  A. Multifaceted approach.
  B. Comforting nursing strategies.
  C. Basic communication strategies.
  D. Teach-back method.

#### 3.3.2. Implications for Patient Results

The overall outcomes derived from the application of communication strategies and nursing relationship styles, as observed in the articles, align with the predetermined criteria outlined in the ScR eligibility guidelines for expected results. In terms of interventions, distinct categories were identified to classify the objectives, thereby consolidating the results obtained from the articles included in the research based on shared themes (Table 6).

1. **Engagement**: refers to the involvement and participation observed after the intervention. Patients become more active, empowered, and responsible.
2. **Decision making**: improves the person’s ability to make, negotiate, or participate in clinical and therapeutic decisions concerning the care pathway/journey.
3. **Costs and safety**: decreased costs due to fewer re-hospitalizations and increased safety.
4. **Satisfaction**: increase in perceived satisfaction resulting from the nursing intervention.
5. **Self-efficacy**: increased effectiveness in communication and interaction.
6. **Health literacy**: increased understanding of information gained and/or increased ability to research it/search for it.
7. **Coping**: redefinition of the concept of health and one’s life trajectory, increased awareness of one’s coping patterns.
8. **Social participation**: helping other people in similar situations within one’s sociocultural context by becoming a point of reference.
9. **Trusting relationship**: maintaining and improving the relationship established with patients.
10. **Self-management**: improvement in symptom management and one’s own treatment.

## 4. Discussion

The examined literature presents various nursing interventions, techniques, and tools that were evaluated from a multidimensional perspective to identify similarities. These findings were subsequently categorized into four clusters, aiming to enhance result comprehension and facilitate understanding of the intricate nature of the subject. The four clusters identified were: generic, specific information and education, tailored to decision making, and supportive–motivational. Despite its specificity, the subject matter encompasses vast and ever-changing research domains, such as relationship and communication, as well as patient engagement. The four clusters are presented in ascending order of complexity. The first appears to be the one which entails generic communication strategies and relational styles. It groups and classifies less complex interventions that require the caregiver to mainly use communication strategies to educate and engage the patient in their own care [36,41,42,47].

The second cluster, namely “Communication strategies and relational styles—specific information and education”, grouped articles related to the development and use of specific communication and relational tools intended to improve the patient’s health literacy, communication, and information skills as well as professional–patient education. Within this cluster, moreover, the use of ICT is particularly observed, which plays an important role in patient engagement. ICTs constitute an interesting area of research and are not considered mere tools to be introduced into the person’s care, but they are new parts of the care pathway that expand and integrate into the already existing and practiced possibilities of relationship, management, surveillance, and communication [39,40,45]. ICTs address the need and desire for transparency and control over personal health information, as well as increasing awareness about one’s health status [8]. Regardless of the number of existing ICTs, all rely on a fundamental aspect between patient and caregiver: trust. Trust is both a cause and effect of ICTs, as it enables the achievement of outcomes from the use of new technologies, such as improving patient involvement and safety, fostering a sense of collaboration [39], enhancing transparency, deepening and managing information exchange (traditionally a caregiver’s task) [49], patient participation, symptom self-management, information sharing, and building trust between caregivers and patients [45]. On the other hand, nurses can communicate more frequently with patients, thereby establishing a supportive relationship that empowers patients to discuss issues that might not have surfaced with any other professional, or recognize over the phone the health status of the patient [45]. Thus, ICTs are not merely tools to be introduced into a person’s care pathway, but new communication strategies and relational styles useful to professionals that expand the available perspectives and practices. 

In the third cluster, “Communication strategies and relational styles—tailored to decision making”, studies highlight the need to share emotions, thoughts, values, ideas, doubts, problems, and needs that the person brings with them in their uniqueness and specificity. This has the characteristic of necessitating the establishment of a deeper relationship rather than, for example, the one developed with the Teach-back method. Furthermore, in this cluster several communication strategies are used within one intervention, which makes these tools even more complex [37,38].

Finally, the last cluster identified is that of “Communication strategies and relational styles—supportive–motivational”. This cluster includes studies with a common characteristic: the presence of tools, relational styles, interventions, and/or techniques to support and/or motivate patients in their empowerment–engagement [15,43,44,46].

**Figure 3:**
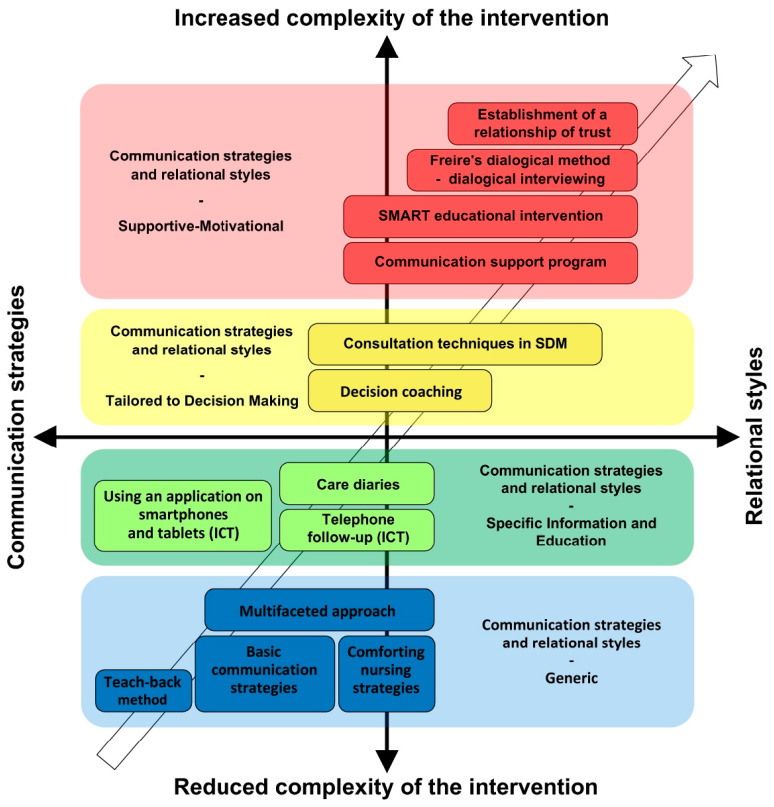
Communicational–relational continuum of the four identified clusters and the interventions tested in the included studies. Legend Figure 3: SMART = specific, measurable, achievable, realistic, timely; SDM = shared decision making; ICT = information and communication technology.

Based on the clusters that were identified, the following diagram (Figure 3) was developed, which allows them to be observed together with the studied interventions extracted from the literature. This diagram shows that as the complexity of the proposed nursing intervention increases, the necessity to establish a relationship with the patient increases and thus not only to use communication strategies but also to integrate specific relational styles into the intervention. Therefore, the axes are to be considered as a continuum, where relationship or communication always coexist as two sides of the same coin.

This pattern is comparable to that described and found in the systematic review conducted by Bonetti et al. [50] with regard to nursing interventions promoting patient engagement in patients with cancer. In particular, it is important to emphasize that the groups of interventions identified in both reviews find commonalities, where the four clusters overlap. Also, with regard to the results, a similarity is observed. For example, results emphasize the importance of the concepts of self-efficacy and health literacy of the person, which are closely related to each other within the complex and articulated world of patient engagement, or, again, the identification of the greater complexity of the supportive–motivational interventions which, according to Bonetti et al. [50], are also the most functional in the promotion of patient engagement.

With reference to the main effects on outcomes that the interventions analyzed had on patients, the results found (direct or indirect) reflected the expected outcomes related to patient engagement and defined in the eligibility criteria (i.e., increased patient satisfaction, patient self-efficacy and patient safety, or decreased costs). Furthermore, outcomes on physiological, psychological, behavioral, and health-related quality of life concerning chronic diseases due to interventions of patient engagement have been significant and consistent with previous studies [51]. This shows that in order to help patients with cancer to participate in their own healthcare it is essential to raise awareness among nurses and other healthcare professionals about implementing patient engagement interventions [1]. In this review, the specific results identified in each article were clustered in turn into ten different categories to highlight the general results that are consequential to communication and relationship tools specifically aimed at the implementation of patient engagement in oncology.

The literature included in the results provides a starting point for future studies in this field, although the extracted articles did not clearly reveal any statistically significant scientific evidence for nursing practice.

This research, however, is not intended to change nursing practice, but it emphasizes the need to pay attention to relations and communication, two powerful nursing tools that nurses use on a daily basis and which can “make a difference”. To achieve the implementation of more complex interventions and to promote patient engagement, in oncology clinical settings it is certainly necessary to raise awareness of the strategies used, the styles implemented, and one’s own competencies regarding communication “basic strategies” and one’s own relational style.

In order to achieve this aim, this ScR mapped the literature to identify knowledge gaps in the chosen topic and to assess the need to develop future research in the field of study under consideration. The proposals are mainly based on two knowledge gaps.

The first consists of the lack of research based on methodological tools to clarify the efficacy and usefulness of communicative and relational nursing techniques, tools, and interventions that have so far only been implemented in specific nursing contexts and/or with research designs that do not statistically prove the benefit of such actions. In fact, most of the articles included in the ScR utilize a primarily qualitative methodology which, by its very nature, has limitations related to the management of the interviews (such as errors caused by the operator during the conduct of the interviews), the diversity of the population included in the various studies (in age, number, culture, and/or social-ethnic groups), the research locations and/or settings (often specific and circumscribed), or, again, the oncological pathologies taken into consideration in the sample or the perceptions of the individual participants who did or did not choose to participate in the study. This implies that the interventions and general results reported in the ScR are not representative of all patients with cancer in all clinical settings, which on the other hand, further clarifies the knowledge gaps in the literature pertaining to the research question. For this reason, it is deemed engrossing to re-evaluate the communication strategies and relational styles identified within this ScR by means of RCTs that aim, for example, to re-evaluate what has been studied and implemented in quality improvement projects.

The second knowledge gap highlighted during the research process, on the other hand, consists in how in the literature there is a certain predisposition to consider and perceive the nurse–patient relationship as a “given” or obviousness in care. Often, in fact, it is understood that this relationship is intrinsic to the nursing profession or as an aspect that is automatically established with every patient during the nursing process. This way of thinking, however, is reductive and simplistic. Indeed, considering this complex and articulated aspect as an interesting topic for research could modify not only clinical practice, but also future nursing training and education, by integrating evaluated interventions that are based on communication and relational skills. Therefore, it becomes essential to plan future research into relational interventions, techniques, and tools that are used or can be implemented in nursing practice. It would be important, on one hand, to clarify the difficulty of establishing a trusting relationship, its benefits, and the more pragmatic aspects such as styles, techniques, tools, and strategies used, and, on the other hand, to investigate which of those is most suitable depending on the clinical, psycho-social profile of the patient. To do so, it is recommended to re-evaluate the tools currently available in the literature, to structure concrete communicative-relational interventions of varying complexity and to introduce them in oncological clinical practice contexts so that RCT studies can be carried out.

## 5. Limitations

The limitations encountered during the search consist of the possible omission of articles relevant to the research question. In fact, the terminology found in the literature regarding the concept of patient engagement is misleading, as some terms are used interchangeably, such as patient participation, patient involvement, patient activation, or even patient empowerment [21,23]. Furthermore, having searched the literature for interventions that necessarily include nurses, although the concept of patient engagement should be considered from an interprofessional perspective, may have resulted in a loss of articles related to specific terms used by other professionals.

## 6. Conclusions

In conclusion, this scoping review shows how relational and communicative nursing interventions are still taken for granted in the literature and considered a “given” of nursing practice. On the contrary, however, studies show the importance of the nurse as a privileged figure in developing patient engagement in adult patients with cancer through their relational communication skills and interventions. Patient engagement, moreover, appears to be an increasingly relevant concept at all levels of healthcare, from the patient’s bedside to national and international healthcare policy choices, and it is for this reason that it is necessary to further broaden knowledge in this area of nursing that enables the evaluation, development, and introduction of interventions, tools, and strategies to promote it. Therefore, researchers should be focusing on identifying “Nursing Communication Strategies and Relational Styles” that are effective and efficient in promoting patient engagement through specific study designs such as RCTs and testing the development of different interventions of varying complexity in daily nursing practice.

## 7. Practice Implications

This study has mapped the literature related to nursing relational and communicative interventions aimed at promoting patient engagement in non-pediatric oncology patients. The ScR has observed the need for increased attention to nurse–patient communication and relationships. Healthcare institutions should invest in training programs and workshops aimed at improving nurse–patient communication skills. This can include active listening, empathetic responses, and effective information-sharing techniques.

The future development of tools and clinical interventions focusing on relational and communicative aspects to promote patient engagement should consider nurses as a fundamental professional figure among a multidisciplinary team. As a consequence, healthcare organizations should acknowledge the central role of nurses in patient engagement within the multidisciplinary team. Nurses should be actively involved in care planning and decision-making processes.

Nurses themselves, in order to engage the patient in their care, must become increasingly aware of the relational and communicative styles they employ in clinical practice, and should be committed to ongoing training. Nurses should engage in self-assessment and self-awareness exercises to identify their communication and relational strengths and weaknesses. Continuous training programs should be offered to enhance these skills.

Incorporating these practice implications can help healthcare institutions and nursing professionals improve patient engagement and ultimately enhance the overall quality of care provided to non-pediatric oncology patients.

## Figures and Tables

**Table 1:** Study characteristics.

Author/s	Year	Title	Country	Aim	Sample	Methodology	Type of Intervention	Outcomes/Main Findings	Key Factors
Birte Berger-Höger, Katrin Liethmann, Ingrid Mühlhauser, Burkhard Haastert, Anke Steckelberg	2019	Nurse-led coaching of shared decision-making for women with ductal carcinoma in situ in breast care centers: A cluster randomized controlled trial	Germany (Certified breast care centers)	The primary objective of the study was to investigate how the proposed intervention improves in patients as well as in caregivers the SDM in relation to therapeutic options and possibilities, allowing an informed evidence-guided choice to be made. The secondary objectives were to assess whether decision-making conflict and medical encounter times are decreased.	192 patients with ductal carcinoma in situ.	Multi-center superiority cluster randomized controlled trial (UK Medical Research Council framework for the development and evaluation of complex interventions).	After training the professionals involved, the interventions implemented compared to the control centers are three: (1) first, a meeting to provide information on the diagnostic therapeutic care pathways and guidance provided to patients by the nurse as early as the first medical visit; (2) a week later, a meeting in which nurse-led decision coaching was implemented, and (3) finally, the last intervention before decision making consists of a meeting with the physician.	The study shows the potential that the use of nurse-led decision coaching has in improving and increasing patient participation in decision making. This communicative-relational technique is evidence-based. Patients’ appreciation of the nurse-led coaching intervention is emphasized in the study. A prerequisite highlighted is the training of nurses in communication and coaching technique.	Decision coaching Communicative and relational tool that allows the patient to be more involved in decision making.
Debbie Bickes, Keisha Jennings, Iris Feinberg	2021	Health Literacy Strategies to Engage Cancer Patients and Caregivers	USA (Cancer hospital network in Georgia—Northside Hospital Cancer Institute)	The objectives of this study were three: the first is to introduce a health literacy intervention among nurse navigators; the second is to evaluate the use of a communicative script during patient interviews; and the third is to evaluate patients’ responses before and after the use of the Teach-back method and thus evaluate the effectiveness and outcomes of the communicative intervention.	55 oncology patients.	Quality improvement study.	At first, the use of the telephone communication method already in use in the institution between navigators and patients was evaluated. The latter required the navigators to ask the patient if he or she had any questions at the end of the interview. Through a survey, the patients’ responses were evaluated. In a second step, based on the results of the evaluation, navigators watched a video on health literacy and reevaluated with an expert the script to be used during the interviews by introducing Teach-back questions. Finally, through two additional surveys, the use of the Teach-back method was evaluated through the responses provided by patients and to clinicians’ perceptions of use.	The results of the study show an increase in patient understanding of the information given and patient engagement, such that the Teach-back method has been adopted as a standardized and routine method of communication in the Hospital Cancer Institute.	Teach-back method Communication method to increase patient engagement: the Teach-back method
Chasity Burrows Walters, Elizabeth Duthie	2017	Patient Engagement as a Patient Safety Strategy: Patients’ Perspectives	USA (National Cancer Institute, Northeastern US cancer center)	This paper aimed to describe patient engagement as a patient safety strategy from the perspective of hospitalized surgical oncology patients.	13 hospitalized surgical oncology patients.	Qualitative approach—grounded theory.	Semi-structured interviews, demographic questionnaires, and the Short Test of Functional Health Literacy in Adults (STOFHLA) were administered. The interviews allowed space for participants to add their views and emerging and unexpected thoughts. Interviews lasted from a minimum of 23 min to a maximum of 64 min and were recorded and transcribed, with the addition of analytical memos by the researcher.	The analysis process highlighted three themes described in the results: the word “patient” obscures the message, safety is a shared responsibility, and being involved in safety is a right. Within these themes, additional sub-themes are described. The results allow the outlining of pragmatic, employable nursing strategies in practice that enable the nurse to facilitate patient engagement, strategies summarized in the Implications for Nursing chapter.	Basic communication strategies Implications for nursing and communication interventions that promote patient engagement are outlined.
Carlin Callaway, Craig Cunningham, Shawna Grover, Kenneth R. Steele, Andrea McGlynn, Vorachai Sribanditmongkol	2018	Patient Handoff Processes	USA (Oncology unit at Naval Medical Center Portsmouth—Virginia)	The study aimed to assess improvement in communication and patient activation, perceived satisfaction and patient activation before and after implementation of the multifaceted approach, and re-admissions before and after implementation of the multifaceted approach. In addition, the study aims to highlight the staff’s experiences regarding the introduced approach.	12 cancer patients; able to make decisions and who were not discharged before 48 h.	Quality improvement project through the use of a multifaceted, evidence-based approach which is structured on the Plan–Do–Study–Act framework.	The project lasted one year, from January 2015 to June 2016. Three interventions were proposed. The first was to introduce handoff at the patient’s bedside, the second to provide discharge information on admission to the hospital, and the third to implement the Teach-back method within the care team. A survey to patients and the care team was administered both before and after the implementation of the multifaceted approach.	The study shows how the implemented multifaceted approach results in decreased hospital re-admissions. In addition, caregivers report a perceived improvement in satisfaction and increased patient engagement (information from anecdotes reported by caregivers).	Multifaceted approach Using three communication and relational methods simultaneously, a multifaceted approach: bedside handoffs, Teach-back method, and discharge bundles that are related to patient engagement.
Li-Chun Chang, I-Chuan Li, Chieh-Hsing Liu	2004	A study of the Empowerment Process for Cancer Patients Using Freire’s Dialogical Interviewing	Taiwan (oncology clinics of a university hospital in Taipei)	The aim of the study was to illustrate the empowering process for cancer patients from the participants’ perspective.	15 patients having had at least 1 week’s elapse since diagnosis of cancer; being alert and capable of verbal communication in Mandarin or Taiwanese.	Qualitative inquiry, through the use of interviews based on Freire’s dialogical method.	The study lasted three months. Patients following radiotherapy or chemotherapy were interviewed. Each person participated in six interviews following which they had 60 to 90 min to write down observations related to the feelings that arose during the interview with the researcher. This information was then discussed at the beginning of the next interview. These notes, moreover, allowed for a record of the interviews, which were not recorded.	The process of empowerment through dialogical interviewing content and process allows the patient to redefine the concept of health and its representation, to be more aware of the coping patterns used and the changes inherent in the disease, to be active and aware of being able to take responsibility, to reevaluate their life trajectory, to negotiate goals of care and discuss/negotiate/decide treatment, and, finally, to help other people in situations similar to their own.	Freire’s dialogical method—dialogical interviewing Communicative interview tool and reflective practice. Establishment of a trusting and supportive relationship.
Yuko Kawasaki	2014	Consultation Techniques Using Shared Decision Making for Patient With Cancer and Their Families	Japan (designated cancer care hospitals (407 locations))	This article elucidates nursing consultation techniques in SDM for patients with cancer and their families.	207 patients with different oncological conditions.	Content analysis involved five stages using the constant comparative method and naturalistic inquiry.	The intervention consists of analysis of the medical records of 207 patients with cancer in which the specialized oncology nurse implemented SDM support. In them, detailed information concerning both the nurse’s interventions and the patients’ responses were described.	The analysis highlighted 8 categories and 24 subcategories of consultation techniques by oncology nurses to support SDM process and decision making support.	Consultation techniques in SDM Eight communicative and relational categories found and helpful in nursing interviews and consultations.
Corinne Rochette, Anne Sophie Michallet, Stéphanie Malartre-Sapienza, Sophie Rodier	2021	Telephone follow-up of oncology patients: the contribution of the nurse specialist for a Service-Dominant Logic in hospital	France (Léon Bérard—cancer control unit)	The aim of this paper is to report how the telephone follow up (by outgoing phone calls) implemented in an oncology department during the active phase of treatment transforms the behaviors of patients.	24 patients (representative of categories present in a previous study from which the population for this study was drawn).	Qualitative approach using semi-structured interviews.	The intervention (November 2018—September 2019) was based on semi-structured interviews with 24 patients drawn from a previously conducted study. The previous study was designed in the following way: during the whole treatment period, 350 patients had received a call twice a week (at predetermined times) from three trained nurses. The call allowed the patients to ask questions and enabled the nurses to understand the development of the clinical situation. The nurse specialist worked closely with the hematologist in order to modify the treatment pathway. At the end of treatment, the intervention through follow-up phone calls also ended.	The article points out that patient telephone follow-up is related to different concepts, including: the establishment of a trusting relationship and its maintenance (following an initial meeting), the convenience and comfort of remote follow-up, the possibility of obtaining information and sharing it, being more active as a patient in one’s own treatment (compliance with drug therapy, self-management of side effects, …), or the possibility of discussing personal aspects that otherwise would not be shared with anyone else (in view of the establishment of the trusting relationship).	Telephone follow-up (ICT) Use of technology communication based on a trusting relationship that enables the implementation of patient activation, research, and information sharing.
Kay Sundberg, Ann Langius Eklöf, Karin Blomberg, Ann-Kristin Isaksson, Yvonne Wengström	2015	Feasibility of an interactive ICT-platform for early assessment and management of patient-reported symptoms during radiotherapy for prostate cancer	Sweden (two university hospitals—one was located in a large city in Sweden and the other in a rural area)	The aim of this study was to test the feasibility and acceptability of an information and communication technology platform for assessing and managing patient-reported symptoms during radiotherapy for prostate cancer.	9 patients with a prostate cancer diagnosis; receiving first-line radiation therapy; being able to read and understand Swedish; and being considered by the clinical team physically, psychologically, and cognitively able to participate in the study.	Patient experience co-design supported by Medical Research Council’s (MRC) complex intervention evaluation framework.	Following the definition of the problem and intervention context (based on the literature review, interviews with patients, and interviews with health professionals), the intervention ICT platform was developed. Then, each patient was equipped with a smartphone after receiving the necessary information regarding the use of the application. For a period of two weeks, the person was asked to track the symptomatology experienced on a daily basis. Finally, interviews were conducted with both patients and nurses involved in the study which were analyzed using the content analysis method.	The use of the smartphone and tablet app by patients with prostate cancer showed improved and increased patient engagement, improved symptom management, easier communication between patients and caregivers, a perceived sense of involvement and collaboration in treatment and with the nurse, satisfaction in rapid and functional interaction with caregivers, and an increased sense of safety resulting from daily symptom surveillance.	Using an app on smartphones and tablets (ICT) Smartphone and tablet app for symptom management in patients with prostate cancer undergoing radiation therapy treatment. Improved communication between patient and caregivers, new communication strategy.
K. Renee Twibell, Debra Siela, Lori Delaney, Patricia Avila, Allison M. Spradlin, Gena Coers	2020	Perspectives of Inpatients With Cancer on Engagement in Fall Prevention	USA (Indiana University Health Ball Memorial Hospital—teaching hospital in Muncie)	The objective of this study was to explore perspectives of hospitalized adults with cancer regarding engagement in fall prevention plans. The primary aim was to discover new knowledge about patients’ perspectives and improve the design of fall prevention strategies. A secondary aim was to compare fall-related perspectives of patients who had and who had not fallen.	30 patients; cognitively alert adults diagnosed with cancer who were at risk for falls (assessed with the John Hopkins Fall Assessment Tool). The inclusion and exclusion criteria are well described in the study. The average age of the participants was 65.4 years, with the youngest being 26 and the oldest 92.	Qualitative descriptive exploratory approach— Semi-structured interviews.	Data collection lasted from 2015 to 2016. The researchers during the interviews highlighted whether participants had been explained their fall risk and tried to understand any thoughts and feelings of the patients regarding the fall prevention plan.	Six themes were identified by the study as representative for patients with cancer in relation to the engagement of fall prevention plans. The themes are as follows: need to get out of bed, decision to call for nursing help, self-assessment and knowledge of self and surrounding spaces, understanding the correlation between cancer pathology and the possibility and consequences of falling, waiting for the required help to arrive, and finally, relationship with nurses.	Establishment of a relationship of trust The study highlights how crucial the patient–nurse relationship is for patients themselves in their engagement in fall prevention programs.
Adam Walczak, Phyllis N. Butow, Martin H.N. Tattersall, Patricia M. Davidson, Jane Young, Ronald M. Epstein, Daniel S.J. Costa, Josephine M. Clayton	2017	Encouraging early discussion of life expectancy and end-of-life care: A randomised controlled trial of a nurse-led communication support program for patients and caregivers	Australia (six cancer treatment centers affiliated with major hospitals in Sydney, Australia)	The objective of this study was to evaluate the effectiveness of the nurse-facilitated communication support program in discussing prognosis and end-of-life care in patients who are in an advanced stage of neoplastic disease characterized by noncurable disease. Four outcomes were sought: increased specific questions in the control group concerning targeted topics of interest to the patient during a follow-up oncology consultation, increased self-efficacy in communicating with the physician, improved involvement in decision making by asserting one’s preferences, and reported improvement in quality of life.	110 patients with advanced, incurable cancer.	Parallel group randomized control trial.	The intervention was based on the implementation of the communication support program (CSP) including a question prompt list (QPL) by experienced nurses who already had good communication skills and were trained for 40 h on the use of this program. The intervention consisted of three phases: the first consists of randomization of the control and intervention groups, the second phase in conducting two audio-recorded CPS sessions (the first live and the second by telephone) of 45 and 15 min, respectively, at a time interval of 1 to 2 weeks after the oncology medical consultation (before and after), and the third in follow-up through the use of various validated questionnaires such as the FACT-G health-related quality-of-life measure to assess the different outcomes researched.	The use of the SPC led the intervention group to have more cues for discussion regarding prognosis, end-of-life care, and possible treatments during the visit with the oncologist, as well as high satisfaction. Self-efficacy in knowing what to ask during the follow-up medical visit was found to be higher in the intervention group than in the control group. The SPC would seem to encourage patients to be more informed regarding their course of treatment.	Communication support program This communication support program turns out to be a strategy that can be employed in nursing practice to engage the patient in decision making and in the care pathway.
Amy Mirabella, Amber Vrana, R. Curtis Bay, Alexandra Slater, Melanie A. Brewer	2022	SMART Oncology Nursing: Literacy, Goals, Coaching and Empowerment	HonorHealth Research and Innovation Institute—Scottsdale, Arizona	The purpose of this study was to evaluate the acceptability and feasibility of structured educational intervention using SMART goals methodology to ameliorate patient outcomes.	68 adults patients (more than 18 years old) with cancer diagnosis (three month prior study enrolment), who speak and read English.	Mixed-methods feasibility.	First of all, a block-randomization by type of cancer was carried out, so as to have two groups: immediate intervention or waitlist control. The educational first group begins intervention, which contains different topics, chosen by the patient with the help of the nurse. Each education module contains instructions, written information, and videos. For each module, two or three SMART objectives are required and for each of these the patient was requested to rate his confidence in being able to successfully complete. At each visit the goals were reviewed. Finally, a follow-up intervention was performed by telephone or in person within one week of the educational intervention. The follow-up intervention allowed answering questions, evaluating the achievement of goals, or carrying out a real-time re-education if necessary. Data were collected from the signed study consent up to 4 weeks after the start of follow-up. Several scales were used in the study: Cancer Health Literacy Test (CHLT-30), 15-item Patient Empowerment Scale (PES), Pearson Separation Index, Adapted Schort Assessment of Patient Satisfaction.	Although not statistically significant, health literacy and patient empowerment increases. Perceived satisfaction also increases. The education intervention, in addition to allowing the establishment of a relationship of trust between patient and nurse, allows the patient to develop skills in order to make health decisions and improve outcomes.	SMART educational intervention Complex tool to develop a relationship of trust, motivate active participation in achieving shared goals, develop health literacy and other concepts related to patient engagement.
Lena Sharp, Göram Laurell, Ylva Tiblom, Arja Andersson, Ros-Marie Birksjö	2004	Care Diaries: A Way of Increasing Head and Neck Cancer Patient’s Involvement in Their Own Care and the Communication Between Clinicians	Department of Otolaryngology and Head & Neck Surgery at Karolinska Hospital and departments of Oncology at Huddinge University Hospital and Karolinska Hospital. Stockholm region, Sweden	The objectives were to collect information, evaluate helpfulness, and discover ways to enhance effectiveness of a care diary, in order to increase patient involvement and increase information among patients, family members, and clinicians.	A total of 120 patients with different cancer typologies used the care diary. Only 66 were considered suitable for answering the questionnaire. A total of 117 questionnaires were collected (42 patients, 28 family members, and 47 clinicians).	Assessment of the care diary—quantitative approach	After a literature search, a qualitative study, and consultation with a multidisciplinary team, the care diary was developed. The latter was subsequently introduced in the three departments involved in the study through information meetings. A nurse “diary coordinator” was trained for each department. The diary was presented to the patient and their family at the first visit to the radiation therapy unit. The nurse, as case manager, provided information on the structure and use of the diary. Ten months after implementation, having reached 120 patients, the questionnaires were sent to the population deemed suitable.	Several outcomes have been identified in the use of the care diary, including: increased satisfaction, feeling important and involved, feeling taken seriously, getting answers to questions and concerns, improved communication, promoted involvement.	Care diaries Care diaries develop participation in one’s own care, and improve communication and relationship with clinicians, patient satisfaction, and other aspects related to patient engagement.
Joan L. Bottorff, Mary Gogag, Michelle Engelberg-Lotykar	1995	Comforting: exploring the work of cancer nurses	Ns (British Columbia University)	The aim of this study was to describe comforting strategies and observe whether they are effective in comforting cancer patients.	Eight cancer patients (3 females and 2 males) during an active treatment cancer ward.	Ethological methods (observational method). A first step saw an inductive descriptive phase, and a second one more structured, deductive, and quantitative investigations.	Every patient was recorded continuously (videotaped) for 72 h. All the interactional units with nurses were identified, isolated, transcribed verbatim and checked for accuracy. The videotaped was analyzed firstly to identify recurring behavior patterns and secondly specific recurring behavioral clusters, and to pay attention also to observed activities as verbal and non-verbal behaviors.	Eight comforting nursing strategies (1. gentle humor, 2. physical comforts, 3. providing information, 4. emotionally supportive statements, 5. choices regarding care, 6. social exchange, 7. increasing proximity, 8. touch) and four patterns of comforting (1. putting experiences into perspective, 2. staying in control, 3. functioning as normally as possible, 4. providing emotional support) were identified. Several benefits have been identified as a result of their use, such as: decreased anxiety, providing informed decision making, active participation in decisions, asking questions.	Comforting nursing strategies Basic communication and interpersonal tools used by the nurse to achieve patient engagement related outcomes, e.g., decreased anxiety and active engagement.

**Table 2:** Interventions and outcomes of quantitative studies.

Authors	Interventions	Outcomes
Berger-Höger et al. (2019) [38]	**Decision coaching** Nurse-led first contact (provide information and written guidelines for understanding the different treatment options). Nurse-led coaching (six steps of shared decision making).	Increases patient participation. Improves decision making. May decrease the cost and time of medical consultations. Appreciation of nursing intervention.
Walczak et al. (2017) [43]	**Communication support program** (using of question prompt list) Patient interviews in preparation for the oncologist interview, followed by follow-up with phone call.	Increased insights for discussion during the visit (increased information and knowledge). Perceived satisfaction higher. Higher self-efficacy in knowing what to ask for.
Sharp et al. (2004) [40]	**Care diaries** The care diary was used and completed by patients, family members, and clinicians in order to record and convey treatment-related information.	Improved satisfaction concerning in the use of the care diary. Feeling involved and important. Feeling taken seriously. Giving answers to questions/doubts. Remembering and repeating information. More effective communication (dedicated communication spaces) and promote improvements in H&N oncological patient care.

**Table 3:** Interventions and results of qualitative studies.

Authors	Interventions	Qualitative Results
Burrows Walters & Duthie (2017) [41]	**Basic communication strategies** Open and direct communication with respect to patient safety (e.g., hand hygiene). Use of signs that reinforce information and education given verbally (e.g., sign reminding people to call for help). Flexibility in understanding how engaged the person wants to be at that point in their care journey and respecting their wishes. Use of appropriate terminology (lay-slang) and provision of clear instructions. Concordance between verbal and body language. Using audio–visual tools or written materials to train the patient.	Increased patient engagement. Internalization of the provided safety message.
Chang et al. (2004) [44]	**Freire’s dialogical method—dialogical interviewing**	Empowerment process. Redefinition of one’s own concept of health. Increased awareness regarding one’s coping patterns. Being more active and more aware of being able to take responsibility. Re-evaluating one’s life trajectory. Negotiating care goals. Deciding, negotiating, and discussing one’s own treatment. Helping others who are in similar situations.
Kawasaki (2014) [37]	**Consultation techniques in SDM** Sharing emotions. Helping to identify key interview points. Helping to personalize one’s plan of care. Providing information in relation to the patient’s needs. Supporting the patient in order to understand the information provided. Ensure continuity of care. Strengthen the support system surrounding the patient.	Supporting patient and family decision making.
Rochette et al. (2021) [45]	**Telephone follow-up (ICT)**	Development and maintenance of a trusting relationship. Increased perceived comfort (e.g., possibility of not having to travel long distances to the hospital). Ability to derive, share information. Increased participation in one’s own treatment (more active in self-management, increased compliance). Confidence to talk about topics that otherwise would not be discussed with anyone (also due to trusting relationship).
Sundberg et al. (2015) [39]	**Use of an app on smartphones and tablets (ICT)**	Improves and increases patient involvement. Improves symptom management. Improves communication. Increases involvement and collaboration in the care pathway and improves the relationship with the nurse. Increases satisfaction since there can be rapid and functional interaction with the nursing staff. Increases safety as there is remote monitoring.
Twibell et al. (2020) [15]	**Establishment of a relationship of trust** Positive nurses interested in the person’s safety. Getting people to perceive authentic caring. Getting to know the patient as a person holistically (e.g., sharing personal stories). Promote compassion and create moments of dialogue. Being honest. Consider the patient as a partner and personalize the safety plan. Use educational videos.	Improves patient engagement. Engages the patient and his/her family in a safety plan.

**Table 4:** Interventions and results of mixed-method studies.

Authors	Interventions	Results
Mirabella et al. (2022) [46]	**SMART educational intervention**	Health literacy increased. Empowerment increased. Perceived satisfaction increased. Living with cancer is not just surviving (change in sick role). Maturing/building a trusting patient–nurse relationship. Making decisions and improving outcomes.
Bottorff et al. (1995) [36]	**Comforting nursing strategies** Gentle humor. Physical comforts. Providing information. Emotionally supportive statements. Choices regarding care. Social exchange. Increasing proximity. Touch. And patterns of comforting Putting experiences into perspective. Staying in control. Functioning as normally as possible. Providing emotional support.	Decrease perceived symptomatology and/or open up to other therapeutic possibilities. Reduce anxiety (to illness/to treatment). Promote informed decision making. Patient actively involved in controlling or modifying the environment. Ask questions about information received; repeat information. Feel free to talk about their experience and feelings. Patient active participation in decisions regarding their personal and nursing care routine. Active involvements in tailoring care to their own needs and desires. Step out of their sick role. Discussing difficult or stressful situations.

**Table 5:** Interventions and results of quality improvement project studies.

Authors	Interventions	Results
Bickes et al. (2021) [47]	**Teach-back method**	Increased patient understanding of the information provided. Increased patient engagement. Adoption of the Teach-back method within the hospital. Empowered patients to be more engaged in their care journey.
Callaway et al. (2018) [42]	**Multifaceted approach** Teach-back method. Bedside handoffs. Discharge bundles.	Decreases in re-hospitalizations. Improved perceived satisfaction. Increased patient engagement.

**Table 6:** General results.

General Results	Specific Results
Engagement	Increased patient participation (Berger-Höger et al., 2019 [38]). Increased patient engagement (Bickes et al., 2021 [47]). Empowered patients to be more engaged in their care journey (Bickes et al., 2021 [47]). Patient actively involved in controlling or modifying the environment (Bottorff et al., 1995 [36]). Increased patient engagement (Burrows Walters & Duthie, 2017 [41]). Increased patient engagement (Callaway et al., 2018 [42]). Empowerment process (Chang L et al., 2004 [44]). More active and empowered patients (Chang L et al., 2004 [44]). Empowerment increased (Mirabella et al., 2022 [46]). Feeling involved and important (Sharp et al., 2004 [40]). Feeling taken seriously (Sharp et al., 2004 [40]). Improved and increased patient involvement (Sundberg et al., 2015 [39]). Increased sense of involvement and collaboration in the care pathway and with the nurse (Sundberg et al., 2015 [39]). Improved patient engagement (Twibell et al., 2020 [15]).
Decision making	Improved decision making (Berger-Höger et al., 2019 [38]). Promote informed decision making (Bottorff et al., 1995 [36]). Patient active participation in decisions regarding their personal and nursing care routine (Bottorff et al., 1995 [36]). Negotiation of treatment goals (Chang et al., 2004 [44]). Decision making, negotiation, and discussion of treatment (Chang et al., 2004 [44]). Ability to support patient and family decision making (Kawasaki, 2014 [37]). Making decisions and improving outcomes (Mirabella et al., 2022 [46]).
Costs and safety	Potential decrease in cost and time of medical consultations (Berger-Höger et al., 2019 [38]). Decreases in re-hospitalizations (Callaway et al., 2018 [42]). Increased safety as there is distance monitoring and surveillance (Sundberg et al., 2015 [39]). Engagement of the patient and their family in a safety plan (Twibell et al., 2020 [15]).
Satisfaction	Appreciation of nursing intervention (Berger-Höger et al., 2019 [38]). Higher perceived satisfaction (Walczak et al., 2017 [43]). Improved perceived satisfaction (Callaway et al., 2018 [42]). Perceived satisfaction increased (Mirabella et al., 2022 [46]). Increased perceived comfort (e.g., possibility of not having to travel long distances to reach the hospital) (Rochette et al., 2021 [45]). Improved satisfaction concerning in the use of the care diary (Sharp et al., 2004 [40]). Increased satisfaction as there can be rapid and functional interaction with caregivers (Sundberg et al., 2015 [39]).
Self-efficacy	Ask questions about information received; repeat information (Bottorff et al., 1995 [36]). Feel free to talk about their experience and feelings (Bottorff et al., 1995 [36]). Discussing difficult or stressful situations (Bottorff et al., 1995 [36]). Giving answers to questions/doubts (Sharp et al., 2004 [40]). Remembering and repeating information (Sharp et al., 2004 [40]). More effective communication (dedicated communication spaces) and promote improvements in H&N oncological patient care (Sharp et al., 2004 [40]). Increased discussion cues during the visit (increased information and knowledge) (Walczak et al., 2017 [43]). Higher self-efficacy in knowing what to ask for (Walczak et al., 2017 [43]). Confidence in talking about topics that otherwise would not be discussed with anyone (also due to trusting relationship) (Rochette et al., 2021 [45]).
Health literacy	Increased patient understanding of the information provided (Bickes et al., 2021 [47]). Adoption of the Teach-back method within (inside) the hospital (Bickes et al., 2021 [47]). Internalization of the message provided about safety (Burrows Walters & Duthie, 2017 [41]). Health literacy increased (Mirabella et al., 2022 [46]). Ability to derive, share information (Rochette et al., 2021 [45]).
Coping	Step out of their sick role (Bottorff et al., 1995 [36]). Redefinition of own concept of health and singular definition (Chang et al., 2004 [44]). Increased awareness with respect to one’s coping patterns (Chang et al., 2004 [44]). Re-evaluation of one’s life trajectory (Chang et al., 2004 [44]). Living with cancer is not just surviving (change in sick role) (Mirabella et al., 2022 [46]).
Social participation	Support for others in similar situations (Chang et al., 2004 [44]).
Trusting relationship	Maturing/building a trusting patient-nurse relationship (Mirabella et al., 2022 [46]). Maintenance and development of a trusting relationship (Rochette et al., 2021 [45]). Improved communication between patient and carer (Sundberg et al., 2015 [39]).
Self-management	Decrease perceived symptomatology and/or open up to other therapeutic possibilities (Bottorff et al., 1995 [36]). Reduce anxiety (to illness/to treatment) (Bottorff et al., 1995 [36]). Active involvements in tailoring care to their own needs and desires (Bottorff et al., 1995 [36]). Increased participation in one’s treatment (more active in self-management, increased compliance) (Rochette et al., 2021 [45]). Improved symptom management (Sundberg et al., 2015 [39]).

## Data Availability

Not applicable.

## Supplementary Materials

The following supporting information can be downloaded at: https://www.mdpi.com/article/10.3390/healthcare12131261/s1, Supplementary File S1. Summary of the database search strategy (complete with the different terms used in the various databases). Supplementary File S2: Search strategy for PUBMED. Supplementary File S3. Full-text articles assessed for eligibility. Supplementary File S4. Quality assessment.
