# Supplementary material for: Uncovering Nursing Communication Strategies and Relational Styles to Foster Patient Engagement in Oncology: A Scoping Review
Source: Healthcare (Basel). 2024 Jun 25;12(13):1261. doi: 10.3390/healthcare12131261 (PMC11241268; doi:10.3390/healthcare12131261)
Supplement: Supplementary file 1 [file healthcare-12-01261-s001.zip › S1. Summary of the database search strategy.docx]

**Supplementary 1**. Summary of the database search strategy (complete with the different terms used in the databases)

The following table summarises the search strategy used in the databases during the advanced search, integrating the logic grid. It highlights the databases used, the combinations created using Boolean operators and finally the total number of articles found. The terms present are subdivided according to PCC.

**Summary of the database search strategy (complete with the different terms used in the various databases)**

| **Database** | **Search strategy** | **Tot. Articles found** |
| --- | --- | --- |
| PubMed (MEDLINE)  CINHAL complete (EBSCO-host)  Cochrane Library  Ovid Nursing Database  APA PsycInfo | \|  \| **Keywords and**  **index terms** \| **Boolean operators** \| \| --- \| --- \| --- \| \| **P** \| “Neoplasms” [MeSH], Tumor, Neoplasm, Tumors, Neoplasia, Neoplasias, Cancer, Cancers, “Malignat Neoplasm”, Malignancy, Malignancies, “Malignant Neoplasms”, “Neoplasm Malignant”, “Neoplasms, Malignant”, “Benign, Neoplasms”, “Benign, Neoplasm”, “Neoplasms, Benign”, “Neoplasm, Benign” \| OR \| \| **C** \| “Nurses” [Mesh], Nurse, “Personnel, Nursing”, “Nursing Personnel”, “Registered Nurses”, “Nurse, Registered”, “Nurses, Registered”, “Registered Nurse”, licensed practical nurse, nurse specialist, nurse training, family nurse practitioner, charge nurse, nurse consultant, advanced practice nurse, nurse anaesthetist, acute care nurse practitioner, nurse researcher, nurse administrator, nurse manager, nurse practitioner, expert nurse, staff nurse, nurse practice act, clinical nurse specialist, adult nurse practitioner, gerontologic nurse practitioner, male nurse, nurse call system, foreign nurse, registered nurse, oncology nurse, practical nurse, head nurse, nurse attitude, emergency nurse practitioner  “Health Communication” [Mesh], “Communication, Health”, “Communications, Health”, “Health Communications”, (MH "Alternative and Augmentative Communication"), (MH "Communication Methods, Total"), exp interpersonal communication/, exp Communication/, exp Communication Skills/  “Nurse-Patient Relations” [Mesh], “Nurse-Patient Relation”, “Relations, Nurse-Patient”, “Nurse Patient Relations”, “Patient Relations, Nurse”, “Relations, Nurse Patient”, “Nurse Patient Relationship”, “Nurse Patient Relationships”, “Patient Relationship, Nurse”, “Patient Relationships, Nurse”, “Relationship, Nurse Patient”, “Relationships, Nurse Patient”, exp Interpersonal Relationships/,  “Patient Participation” [Mesh], “Participation, Patient”, “Patient Involvement”, “Involvement, Patient”, “Patient Empowerment”, “Empowerment, Patient”, “Patient Participation Rates”, “Participation Rate, Patient”, “Participation Rates, Patient”, “Patient Participation Rate”, “Patient Activation”, “Activation, Patient”, “Patient Engagement”, “Engagement, Patient”, (MH “Consumer Participation”), “consumer participation”, “consumer engagement”, exp Client Participation/ \| AND  OR  OR  AND  OR  OR  OR \| \| **C** \| No limits \|  \| | 1899 |
