# Supplementary material for: Uncovering Nursing Communication Strategies and Relational Styles to Foster Patient Engagement in Oncology: A Scoping Review
Source: Healthcare (Basel). 2024 Jun 25;12(13):1261. doi: 10.3390/healthcare12131261 (PMC11241268; doi:10.3390/healthcare12131261)
Supplement: Supplementary file 1 [file healthcare-12-01261-s001.zip › S2. Search strategy.docx]

**Supplementary 2**. Search strategy

**PUBMED SEARCH STRATEGY**

| **#26** | **Add** | **(((((“Patient Participation” [Mesh]) OR (“Participation, Patient” OR “Patient Involvement” OR “Involvement, Patient” OR “Patient Empowerment” OR “Empowerment, Patient” OR “Patient Participation Rates” OR “Participation Rate, Patient” OR “Participation Rates, Patient” OR “Patient Participation Rate” OR “Patient Activation” OR “Activation, Patient” OR “Patient Engagement” OR “Engagement, Patient”)))) AND (((“Nurses” [Mesh]) OR (Nurse OR “Personnel, Nursing” OR “Nursing Personnel” OR “Registered Nurses” OR “Nurse, Registered” OR “Nurses, Registered” OR “Registered Nurse”)) OR ((“Health Communication” [Mesh]) OR (“Communication, Health” OR “Communications, Health” OR “Health Communications”)) OR ((“Nurse-Patient Relations” [Mesh]) OR (“Nurse-Patient Relation” OR “Relations, Nurse-Patient” OR “Nurse Patient Relations” OR “Patient Relations, Nurse” OR “Relations, Nurse Patient” OR “Nurse Patient Relationship” OR “Nurse Patient Relationships” OR “Patient Relationship, Nurse” OR “Patient Relationships, Nurse” OR “Relationship, Nurse Patient” OR “Relationships, Nurse Patient”))) AND ((“Neoplasms” [MeSH]) OR (Tumor OR Neoplasm OR Tumors OR Neoplasia OR Neoplasias OR Cancer OR Cancers OR “Malignat Neoplasm” OR Malignancy OR Malignancies OR “Malignant Neoplasms” OR “Neoplasm, Malignant” OR “Neoplasms, Malignant” OR “Benign, Neoplasms” OR “Benign, Neoplasm” OR “Neoplasms, Benign” OR “Neoplasm, Benign”))** | **455** |
| --- | --- | --- | --- |
| #24 | Add | (((“Nurses” [Mesh]) OR (Nurse OR “Personnel, Nursing” OR “Nursing Personnel” OR “Registered Nurses” OR “Nurse, Registered” OR “Nurses, Registered” OR “Registered Nurse”)) OR ((“Health Communication” [Mesh]) OR (“Communication, Health” OR “Communications, Health” OR “Health Communications”)) OR ((“Nurse-Patient Relations” [Mesh]) OR (“Nurse-Patient Relation” OR “Relations, Nurse-Patient” OR “Nurse Patient Relations” OR “Patient Relations, Nurse” OR “Relations, Nurse Patient” OR “Nurse Patient Relationship” OR “Nurse Patient Relationships” OR “Patient Relationship, Nurse” OR “Patient Relationships, Nurse” OR “Relationship, Nurse Patient” OR “Relationships, Nurse Patient”))) | 430’647 |
| #23 | Add | (“Health Communication” [Mesh]) OR (“Communication, Health” OR “Communications, Health” OR “Health Communications”) | 3’718 |
| #22 | Add | (“Nurse-Patient Relations” [Mesh]) OR (“Nurse-Patient Relation” OR “Relations, Nurse-Patient” OR “Nurse Patient Relations” OR “Patient Relations, Nurse” OR “Relations, Nurse Patient” OR “Nurse Patient Relationship” OR “Nurse Patient Relationships” OR “Patient Relationship, Nurse” OR “Patient Relationships, Nurse” OR “Relationship, Nurse Patient” OR “Relationships, Nurse Patient”) | 38’642 |
| #21 | Add | (“Patient Participation” [Mesh]) OR (“Participation, Patient” OR “Patient Involvement” OR “Involvement, Patient” OR “Patient Empowerment” OR “Empowerment, Patient” OR “Patient Participation Rates” OR “Participation Rate, Patient” OR “Participation Rates, Patient” OR “Patient Participation Rate” OR “Patient Activation” OR “Activation, Patient” OR “Patient Engagement” OR “Engagement, Patient”) | 34’957 |
| #20 | Add | (“Nurses” [Mesh]) OR (Nurse OR “Personnel, Nursing” OR “Nursing Personnel” OR “Registered Nurses” OR “Nurse, Registered” OR “Nurses, Registered” OR “Registered Nurse”) | 410’281 |
| #19 | Add | (“Neoplasms” [MeSH]) OR (Tumor OR Neoplasm OR Tumors OR Neoplasia OR Neoplasias OR Cancer OR Cancers OR “Malignat Neoplasm” OR Malignancy OR Malignancies OR “Malignant Neoplasms” OR “Neoplasm, Malignant” OR “Neoplasms, Malignant” OR “Benign, Neoplasms” OR “Benign, Neoplasm” OR “Neoplasms, Benign” OR “Neoplasm, Benign”) | 5’176’648 |
| #15 |  | “Communication, Health” OR “Communications, Health” OR “Health Communications” | 830 |
| #14 |  | “Health Communication” [Mesh] | 2’982 |
| #13 |  | “Nurse-Patient Relation” OR “Relations, Nurse-Patient” OR “Nurse Patient Relations” OR “Patient Relations, Nurse” OR “Relations, Nurse Patient” OR “Nurse Patient Relationship” OR “Nurse Patient Relationships” OR “Patient Relationship, Nurse” OR “Patient Relationships, Nurse” OR “Relationship, Nurse Patient” OR “Relationships, Nurse Patient” | 38’642 |
| #12 |  | “Nurse-Patient Relations” [Mesh] | 35’921 |
| #11 |  | “Participation, Patient” OR “Patient Involvement” OR “Involvement, Patient” OR “Patient Empowerment” OR “Empowerment, Patient” OR “Patient Participation Rates” OR “Participation Rate, Patient” OR “Participation Rates, Patient” OR “Patient Participation Rate” OR “Patient Activation” OR “Activation, Patient” OR “Patient Engagement” OR “Engagement, Patient” | 9’829 |
| #9 |  | “Patient Participation” [Mesh] | 28’169 |
| #5 |  | Nurse OR “Personnel, Nursing” OR “Nursing Personnel” OR “Registered Nurses” OR “Nurse, Registered” OR “Nurses, Registered” OR “Registered Nurse” | 410’281 |
| #4 |  | “Nurses” [Mesh] | 94’461 |
| #2 |  | Tumor OR Neoplasm OR Tumors OR Neoplasia OR Neoplasias OR Cancer OR Cancers OR “Malignat Neoplasm” OR Malignancy OR Malignancies OR “Malignant Neoplasms” OR “Neoplasm, Malignant” OR “Neoplasms, Malignant” OR “Benign, Neoplasms” OR “Benign, Neoplasm” OR “Neoplasms, Benign” OR “Neoplasm, Benign” | 5'176’648 |
| #1 |  | Search  “Neoplasms” [MeSH] | 3'609’601 |
| **Development of advanced search strings** | | | |
| #7 |  | (((Neoplasia OR Cancer OR “Cancer care” OR “Cancer patient” OR “Oncology patient” OR Tumor AND Communication OR “Health communication” OR “Patient-provider communication” OR Relationship OR “Nurse-patient relations” OR “Nurse-patient relationship”) AND (Nurse OR Nursing OR “Oncology nursing”)) AND (“Patient engagement” OR Engagement OR “Patient participation” OR “Patient empowerment” OR “Patient partnership” OR “Patient-centeredness” OR “Patient activation” OR “Patient involvement”) | 7’339 |
| #5 | Add | (((Cancer OR Oncology patient) AND (Communication OR Relationship OR Nurse-patient relations OR Nurse-patient relationship) AND (Nurse OR Nursing OR Oncology nursing)) AND (Patient engagement OR Patient participation OR Patient partnership OR Patient activation OR Patient involvement)) | 1’097 |
| #1 | Add | Cancer AND (Communication OR Relationship) AND Nurse AND “Patient engagement” | 15 |
| **Free-form initial search strings** | | | |
