# Supplementary material for: Uncovering Nursing Communication Strategies and Relational Styles to Foster Patient Engagement in Oncology: A Scoping Review
Source: Healthcare (Basel). 2024 Jun 25;12(13):1261. doi: 10.3390/healthcare12131261 (PMC11241268; doi:10.3390/healthcare12131261)
Supplement: Supplementary file 1 [file healthcare-12-01261-s001.zip › S3. Full-text articles assessed for eligibility.docx]

**Supplementary 3.** Full-text articles assessed for eligibility

**71 full-text articles assessed for eligibility**

|  | **Author - year of publication** | **Included** | **Excluded** | **Reason for exclusion** |
| --- | --- | --- | --- | --- |
| 1 | Al-Itejawi et al. - 2016 |  | X | Does not outline any relational or communicative nursing intervention. |
| 2 | Allen e Berry - 2011 |  | X | Does not outline any relational or communicative nursing intervention. |
| 3 | Anderson AS e Klemm P – 2008 |  | X | Educational intervention of the nurse; no correlation to patient engagement. |
| 4 | Ankolekar et al. - 2019 |  | X | Highlights Share Decision-making without discussing relational and communicative nursing interventions. |
| 5 | Arora - 2013 |  | X | Does not outline any relational or communicative nursing intervention. |
| 6 | Beaumont & Crawford-Gray – 2016 |  | X | Methodology is not part of the inclusion criteria. |
| 7 | Beisecker et al. - 1994 |  | X | Does not outline any relational or communicative nursing intervention. |
| 8 | Berger-Höger et al. - 2019 | X |  |  |
| 9 | Bellomo et al. – 2019 |  | X | Methodology is not part of the inclusion criteria. |
| 10 | Bickes et al. - 2021 | X |  |  |
| 11 | Boman et al. – 2018 |  | X | Does not outline any relational or communicative nursing intervention. |
| 12 | Boonzaier et al. – 2009 |  | X | Methodology is not part of the inclusion criteria (*Pilot study).* |
| 13 | Bottorff et al. - 1995 | X |  |  |
| 14 | Burrows Walters e Duthie - 2017 | X |  |  |
| 15 | Callaway et al. - 2018 | X |  |  |
| 16 | Chabrera et al. - 2015 |  | X | Does not outline any relational or communicative nursing intervention. |
| 17 | Chang et al. - 2004 | X |  |  |
| 18 | Clarke et al. - 2020 |  | X | Methodology is not part of the inclusion criteria. |
| 19 | Colella KM e DeLuca G - 2004 |  | X | Methodology is not part of the inclusion criteria (*Pilot study).* |
| 20 | Coleman - 2014 |  | X | Methodology is not part of the inclusion criteria. |
| 21 | Davids et al. – n.d. |  | X | Does not outline any relational or communicative nursing intervention. |
| 22 | Davis et al. – 2011 |  | X | Population not only includes cancer patients. |
| 23 | de Veer et al. - 2020 |  | X | Does not outline any relational or communicative nursing intervention. |
| 24 | Eddleman & Warren - 1994 |  | X | Does not outline any relational or communicative nursing intervention. |
| 25 | Eun-Shim Nahm et al. - 2019 |  | X | Methodology is not part of the inclusion criteria (P*ilot study).* |
| 26 | Faiman e Tariman - 2019 |  | X | Does not outline any relational or communicative nursing intervention.  It highlights other non-relational non-communicative interventions. |
| 27 | Fleisher et al. - 2015 |  | X | Missing nurse population. |
| 28 | Hibbard et al. - 2017 |  | X | Does not outline any relational or communicative nursing intervention. |
| 29 | Hogberg et al. – 2015 |  | X | Methodology is not part of the inclusion criteria (*Pilot study)*.  Population including not only cancer patients. |
| 30 | Jenerette e Mayer - 2016 |  | X | Methodology is not part of the inclusion criteria. |
| 31 | Jerofke - 2013 |  | X | Methodology is not part of the inclusion criteria. |
| 32 | Jeyathevan et al. – 2017 |  | X | Does not outline any relational or communicative nursing intervention.  It highlights other non-relational non-communicative interventions. |
| 33 | Kawasaki – 2014 | X |  |  |
| 34 | Kottschade e Reed - 2017 |  | X | Methodology is not part of the inclusion criteria. |
| 35 | Kullberg et al. - 2015 |  | X | It states that communication increases patient satisfaction, participation and safety but does not give specifics. Comparison with MD according to patient rating scale. |
| 36 | Kullberg et al. - 2018 |  | x | Population includes only nurses and not patients. |
| 37 | LeBlanc - 2019 |  | X | Methodology is not part of the inclusion criteria. |
| 38 | Lin et al. - 2019 |  | X | The importance of communication between patient and clinician is emphasised but no specific communication strategies or relational styles are outlined. |
| 39 | Lum et al. - 1978 |  |  | Does not outline any relational or communicative nursing intervention. |
| 40 | Mayer - 2014 |  | X | Methodology is not part of the inclusion criteria. |
| 41 | Marks et al. - 2010 |  | X | Does not outline any relational or communicative nursing intervention.  Missing nurse population. |
| 42 | Mccullough et al. - 2010 |  | X | Does not outline any relational or communicative nursing intervention.  It recognises the importance of communication and emphasises its necessity in nursing education. |
| 43 | Millard et al. – 2006 |  | X | Population not only includes cancer patients. |
| 44 | Mirabella et al. – 2022 | X |  |  |
| 45 | Mok - 2001 |  | X | Does not outline any relational or communicative nursing intervention. |
| 46 | Perfors et al. – 2018i |  | X | Methodology is not part of the inclusion criteria (research protocol). |
| 47 | Pongthavornkamol et al. - 2018 |  | X | It emphasises the importance of communication and relationship in patient participation but does not outline communication strategies and relationship styles. |
| 48 | Post et al. – 2021 |  | X | Does not outline any relational or communicative nursing intervention. |
| 49 | Qaderi et al. - 2020 |  | X | Does not outline any relational or communicative nursing intervention. |
| 50 | Rochette et al. - 2021 | X |  |  |
| 51 | Rocque et al. - 2018 |  | X | Methodology is not part of the inclusion criteria (*Pilot study).* |
| 52 | Rodriguez - 2018 |  | X | Methodology is not part of the inclusion criteria. |
| 53 | Rosales et al. - 2014 |  | X | Does not outline any relational or communicative nursing intervention. |
| 54 | Rustøen & Schjølberg - 2000 |  | X | Does not outline any relational or communicative nursing intervention.  Also paediatric population. |
| 55 | Sainio et al. - 2001 |  | X | Does not outline any relational or communicative nursing intervention. |
| 56 | Scharp et al. - 2004 | X |  |  |
| 57 | Schwappach DLB et al. - 2010 |  | X | Does not outline any relational or communicative nursing intervention. |
| 58 | Schwappach e Wernli - 2010 |  | X | Does not outline any relational or communicative nursing intervention. |
| 59 | Sepucha KR et al. - 2003 |  | X | Methodology is not part of the inclusion criteria. |
| 60 | Stacey et al. - 2008 |  | X | Does not outline any relational or communicative nursing intervention. |
| 61 | Stacey et al. - 2020 |  | X | Does not outline any relational or communicative nursing intervention. |
| 62 | Sundberg et al. - 2015 | X |  |  |
| 63 | Suomien – n.d. |  | X | Methodology is not part of the inclusion criteria. |
| 64 | Suominen et al. - 1994 |  | X | Does not outline any relational or communicative nursing intervention. |
| 65 | Tariman e Szubski - 2015 |  | X | Does not outline any relational or communicative nursing intervention. |
| 66 | Tariman et al. - 2014 |  | X | Does not outline any relational or communicative nursing intervention. |
| 67 | Twibell et al. – 2020 | X |  |  |
| 68 | Walczak et al. – 2017 | X |  |  |
| 69 | Wilfong et al. – 2016 |  | X | Methodology is not part of the inclusion criteria. |
| 70 | Wilson e Mooney – 2020 |  | X | Does not outline any relational or communicative nursing intervention. |
| 71 | Witte e Handberg – 2019 |  | X | Does not outline any relational or communicative nursing intervention. |
