# Supplementary material for: Uncovering Nursing Communication Strategies and Relational Styles to Foster Patient Engagement in Oncology: A Scoping Review
Source: Healthcare (Basel). 2024 Jun 25;12(13):1261. doi: 10.3390/healthcare12131261 (PMC11241268; doi:10.3390/healthcare12131261)
Supplement: Supplementary file 1 [file healthcare-12-01261-s001.zip › S4. Quality assessment.docx]

Supplementary 4: Quality assessment

| Articles | 1. Abstract and title: Did they provide a clear description of the study? | 2. Introduction and aims: Was there a good background and clear statement of the aims of the research? | 3. Method and data: Is the method appropriate and clearly explained? | 4. Sampling: Was the sampling strategy appropriate to address the aims? | 5. Data analysis: Was the description of the data analysis sufficiently rigorous? | 6. Ethics and bias: Have ethical issues been addressed, and what has necessary ethical approval gained? Has the relationship between researchers and partecipiants been adequately considered? | 7. Results: Is there a clear statement of the findings? | 8. Transferability or generalizability: Are the findings of this study transferable (generalizable) to a wider population? | 9. Implications and usefulness: How important are these findings to policy and practice? | NOTE |
| --- | --- | --- | --- | --- | --- | --- | --- | --- | --- | --- |
| **Quantitative studies** | | | | | | | | | | |
| Berger-Höger et al. (2019) | Good | Good | Good | Good | Good | Good | Good | Good | Good |  |
| Walczak et al. (2017) | Good | Good | Good | Good | Good | Good | Good | Good | Good |  |
| Sharp et al. (2004) | Good | Good | Good | Good | Fair | Good | Good | Good | Good |  |
| **Qualitative studies** | | | | | | | | | | |
| Burrows Walters & Duthie (2017) | Good | Good | Good | Good | Good | Good | Fair | Good | Good |  |
| Chang et al. (2004) | Good | Good | Good | Good | Good | Poor | Good | Fair | Good |  |
| Kawasaki (2014) | Good | Fair | Fair | Good | Good | Good | Good | Fair | Good |  |
| Rochette et al. (2021) | Good | Good | Good | Good | Good | Fair | Good | Good | Good |  |
| Sundberg et al. (2015) | Good | Good | Good | Good | Good | Fair | Good | Good | Good |  |
| Twibell et al. (2020) | Good | Good | Good | Good | Good | Good | Good | Good | Good |  |
| **Mixed method studies** | | | | | | | | | | |
| Mirabella et al. (2022) | Fair | Good | Good | Good | Good | Fair | Good | Good | Good |  |
| Bottorff et al. (1995) | Good | Fair | Good | Poor | Poor | Fair | Good | Poor | Fair | Missing: Suggests ideas for further research |
| **Quality improvement project studies** | | | | | | | | | | |
| Bickes et al. (2021) | Good | Fair | Fair | Poor | Good | Poor | Fair | Poor | Fair | Missing: Suggests ideas for further research |
| Callaway et al. (2018) | Good | Good | Good | Poor | Good | Fair | Fair? Good | Poor | Good |  |

Legenda: Very Poor / Poor / Fair / Good
